# Family Doctor Contract Services and Awareness of Blood Pressure Measurement Among Hypertension Patients: A Cross-Sectional Study in Rural Shandong, China

**DOI:** 10.3389/fpubh.2022.757481

**Published:** 2022-03-16

**Authors:** Chen Yan, Yemin Yuan, Dan Zhao, Jie Li, Peipei Fu, Yan Chen, Jie Li, Zhixian Li, Shijun Yang, Wenjuan Li, Zhen Gui, Xiuqing Peng, Chengchao Zhou

**Affiliations:** ^1^Centre for Health Management and Policy Research, School of Public Health, Cheeloo College of Medicine, Shandong University, Jinan, China; ^2^National Health Committee (NHC) Key Lab of Health Economics and Policy Research, Shandong University, Jinan, China; ^3^Department of Epidemiology, School of Public Health, Cheeloo College of Medicine, Shandong University, Jinan, China; ^4^School of Public Health, Wannan Medical College, Wuhu, China; ^5^Shandong Provincial Hospital Affiliated to Shandong First Medical University, Jinan, China

**Keywords:** primary care, hypertension, family doctor contract services, awareness, China

## Abstract

**Background:**

Some studies found that family doctor contract services (FDCSs) had positive impact on the self-measurement behaviors of hypertension patients. However, evidence concerning the association between FDCSs and blood pressure measurement awareness among hypertension patients is not clear.

**Objective:**

This study aims to explore the relationship between FDCSs and blood pressure measurement awareness among the hypertension patients, and examine whether there is a difference in this relationship among middle-aged and aged adults in rural Shandong, China.

**Methods:**

A multi-stage stratified random sampling was adopted in 2018 in Shandong Province to conduct a questionnaire survey among the sample residents, in which 982 hypertension patients were included in the study. Pearson chi-square test and logistic regression model were employed using SPSS 24.0 to explore the association between FDCSs and blood pressure measurement awareness.

**Results:**

76.8% of hypertension patients would measure blood pressure regularly. The blood pressure measurement awareness of the signing group was significantly higher than that of the non-signing group when controlling other variables (*P* < 0.001, *OR* = 2.075, 95% *CI* 1.391–3.095). The interaction of age and contracting status were significantly correlated with blood pressure measurement awareness (*P* = 0.042, *OR* = 1.747, 95% *CI* 1.020–2.992; *P* = 0.019, *OR* = 2.060, 95% *CI* 1.129–3.759). Factors including gender (*P* = 0.011, *OR* = 0.499, 95% *CI* 0.291–0.855), household income (*P* = 0.031, *OR* = 1.764, 95% *CI* 1.052–2.956), smoking status (*P* = 0.002, *OR* = 0.439, 95% *CI* 0.260–0.739), sports habits (*P* < 0.001, *OR* = 2.338, 95% *CI* 1.679–3.257), self-reported health (*P* = 0.031, *OR* = 1.608, 95% *CI* 1.043–2.477), distance to the village clinic (*P* = 0.006, *OR* = 1.952, 95% *CI* 1.208–3.153) and medications (*P* < 0.001, *OR* = 3.345, 95% *CI* 2.282–4.904) were also found to be associated with the blood pressure measurement awareness of hypertension patients.

**Conclusion:**

The government should take efforts to strengthen publicity and education of family doctors and pay more attention to uncontracted, middle-aged, female patients and patients with unhealthy life behaviors to improve the blood pressure measurement awareness.

## Introduction

The prevalence of hypertension increases with age (1), causes 7.5 million deaths per year (12.8% of all death) (2) and is a growing public health problem globally (3). In China, about a third of adults suffer from high blood pressure (4). The prevalence of hypertension is still on the rise in China, and this prevalence in rural residents increases faster than that in urban areas (5). Hypertension is a risk factor for many serious diseases, such as stroke, intracerebral hemorrhage, which places a heavy burden on the patients, their families, and even the whole health system (6). Thus, effective control and management is crucial for hypertension in China.

In the management of hypertension in China, family doctor contract services (FDCSs) plays an important role. China proposed the establishment of a general practitioner (GP) system to carry out contract services in 2011, which has strengthened the importance of primary care centers in the national health strategy (7). FDCSs are essentially the extension and development of community health services, providing a proactive, continuous, and comprehensive health accountability management model by signing service contracts with residents (8). FDCSs include regular monitoring, health profile updates, and health education and promotion, etc. (9). Among them, hypertension management of patients is the basic project, which carries out work from psychological, physical, diet, exercise and other aspects (10). Hypertension measurement is a key item which help patients know their blood pressure (11).

In addition to the measurement of hypertension by family doctors, self-measurement is more important. Family doctors can develop good self-measurement behaviors by cultivating awareness of blood pressure measurement to help patients master health status. The Chinese Guidelines for the Prevention and Treatment of Hypertension emphasizes the effect of health awareness on hypertension management (5). Patients' management and measurement awareness are important predictor of management adherence and optimal blood pressure control (12–15). Blood pressure measurement awareness is crucial to control and treat hypertension, and is important for the hypertension individuals to change their unhealthy lifestyle and dietary habits (16). Some studies found that there was a positive association between blood pressure management awareness and hypertension management and adherence to antihypertensive treatment (17–19). However, most previous studies have focused on improving hypertension self-management behaviors (20), but blood pressure measurement awareness was paid little attention to. Awareness of blood pressure measurement is the foundation of self-management behavior. In order to further improve blood pressure control effect, to understand the blood pressure measurement awareness and its characteristics among hypertension patients is of high priority (16).

Studies on measurement awareness of hypertension differ across different regions globally. A cross-sectional analysis using data from the CONSTANCES population-based cohort in France suggested that patients' hypertension awareness was low and effective action was required to improve hypertension awareness (16). Another study in Korea found that self-blood pressure monitoring from hospitals was associated with improved awareness of blood pressure among the patients (21). In China, the awareness rate of hypertension patients was low, which would result in a potential huge burden of cardiovascular disease in the future (22). Community health management is helpful to strengthen the prevention and treatment awareness of hypertension patients, reduce the incidence and death of cardiovascular diseases to the maximum extent (23). In addition, age was an influencing factor for hypertension patients to participate in health management (24).

However, to date, there are very few studies to explore the association between FDCSs and residents' hypertension self-measurement awareness in China. To remedy this gap, the current study aims to explore whether being contracted with a family doctor is associated with blood pressure measurement awareness in rural Shandong, China. The specific objectives of this study are: (1) to describe the rate of blood pressure measurement awareness among the hypertension patients; (2) to explore the relationship between FDCSs and blood pressure measurement awareness among middle-aged and aged hypertension patients, and other related factors.

## Methods

### Sampling

This study was conducted in 2018 in Shandong Province, the second largest province in China. The sample size calculation was performed by the following formula:


$$\begin{array}{l} N=\frac{u_{\propto /2}^{2}\pi \left(1-\pi \right)}{\delta^{2}}\left(\pi :\text{expected prevalence}\right) \end{array}$$

The prevalence of hypertension awareness among Chinese residents was 46.9% according to previous studies (22), for reference, in this study, π= 0.47, *u*_α/2_= 1.96, δ= 0.1 π= 0.047, α = 0.05. We found that required sample size was 443. Taking into account the loss and refusal of interviews, the final sample size of the survey was determined to be 500.

A multistage stratified random sampling method was used to select the participants. First, three cities—Binzhou, Zibo, and Liaocheng located in the northeast, central, and west of Shandong Province, which represented the medium, high, and low levels of economic status according to the GDP per capita (2018), respectively. Second, two counties in each city were firstly chosen at random. Within each county, five townships and five villages in each township were randomly chosen. Eighteen households were randomly selected from each village. To ensure the randomness of the sample, the research team provided the names of all the towns in the sample counties, and commissioned experts from the School of Public Health at the University of North Carolina at Chapel Hill, who had maintained a long-term cooperative relationship, to select the sample towns using a random number table. The investigators were recruited from Shandong University. They were strictly trained before the investigation, including understanding the principles and methods of the survey, and standardizing the definition and interview skills of each study indicator, with the purpose of ensuring the quality of the survey. All the participants were interviewed face-to-face using a structured questionnaire by trained investigators. Completed questionnaires were carefully checked by the supervisors after the interview each day. In total, 2,979 individuals were recruited and completed the whole survey.

The diagnosis of hypertension was confirmed by the Chinese Guidelines for the Prevention and Treatment of Hypertension in which hypertension was SBP ≥140 mmHg and/or DBP ≥90 mmHg in the absence of antihypertensive drugs (5). Hypertension conditions were self-reported. To validate the accuracy of this information, the trained interviewers with medical knowledge would further ask the help from the village doctors to confirm the information of self-reported chronic conditions in the system of chronic disease case management in the sampling villages. Nine hundred eighty-two adults diagnosed with hypertension were included in the study. Participants were divided into the middle-aged group and the elderly group based on the cut-off point of 65 (25) years for analysis.

### Measurement

#### Blood Pressure Measurement Awareness

The blood pressure measurement awareness in this study was measured by the following question: “Do you think you need to measure your blood pressure regularly [test yourself at least 1 day a week (5)]?” and the hypertension patients could respond with “yes” (coded as 1) or “no” (coded as 0). Participants with hypertension would answer this question.

#### Family Doctor Contracting Status

The family doctor contracting status of the respondents was measured by the following question: “Did you contract with the family doctors this year?,” they could respond with “yes” (coded as 1) or “no” (coded as 0).

#### Covariate Variables

We identified the potential confounders on the basis of the existing studies (2, 26), including gender, educational level (illiteracy, primary or below, and secondary school or above), household income (Quartile 1 was the poorest and Quartile 4 was the richest), smoking status (current vs. never/past), alcohol drinking status (current vs. never/past), self-reported health status (very healthy/healthy, general and unhealthy/very unhealthy), sport activities (current vs. never/past), distance to the village clinic (meters, ≤100, 101–500, >500), multiple chronic diseases (include diabetes and coronary artery disease) and medications (have you taken hypertension medication in the past 2 weeks).

### Ethical Considerations

This study was approved by the Ethical Committee of Shandong University School of Public Health. All participants provided informed consent before the face-to-face interview. The data used in this study were anonymized before use.

### Statistical Analysis

The data were double-entered and coded using EpiData version 3.1, and were analyzed using IBM SPSS 24.0 (Inc., Chicago, Illinois, USA). All study variables were performed with a descriptive analysis. Pearson chi-square tests were performed to assess whether socio-demographic variables, family doctor contracting status, and other factors were related to awareness of blood pressure measurement. Binary logistic regression model was employed to explore association between family doctor contracting status and blood pressure management awareness, adjusting for gender, educational level, household income per year, smoking status, drinking status, self-reported health, distance to the village clinic, multiple chronic diseases and medications. We also estimated the 95% confidence interval of OR. All tests were two-tailed and a *p*-value of <0.05 was considered statistically significant.

## Results

### Socio-Demographic Characteristics of the Participants

Table 1 presents the characteristics of the sample and the differences of blood pressure measurement awareness. Among 982 participants, 443 (45.11%) were male and 539 (54.89%) were female. 521 (53.05%) were middle-aged residents and 461 (46.95%) were aged residents, and 754 (76.78%) would measure blood pressure regularly. The majority of the respondents were primary school degree or below (34.01%), never smokers (68.23%), never drinkers (63.54%), without multiple chronic diseases (66.09%). 813 (82.79%) of them took hypertension medications in the last 2 weeks.

**Table 1:** Description and signal analysis of blood pressure measurement awareness among hypertension patients in rural Shandong, China (*N* = 982).

**Characteristics**	***N* (%)**	**Hypertension measurement awareness**		***x* ^2^**	***P*-value**
		**No [*N* (%)]**	**Yes [*N* (%)]**		
***N*** **(%)**	982 (100.00)	228 (23.22)	754 (76.78)		
**Gender**				0.791	0.374
Male	443 (45.11)	97 (21.90)	346 (78.10)		
Female	539 (54.89)	131 (24.30)	408 (75.70)		
**Age**				0.025	0.875
Middle-aged (<65)	521 (53.05)	122 (23.42)	399 (76.58)		
Aged (≥65)	461 (46.95)	106 (22.99)	355 (77.01)		
**Education level**				2.667	0.264
Illiteracy	320 (32.59)	80 (25.00)	240 (75.00)		
Primary or below	334 (34.01)	82 (24.55)	252 (75.45)		
Secondary school or above	328 (33.40)	66 (20.12)	262 (79.88)		
**Household income per year (RMB, Yuan)**				9.000	0.029
Q1 (≤ 8,400)	251 (25.56)	60 (23.90)	191 (76.10)		
Q2 (8,401–20,000)	259 (26.37)	73 (28.19)	186 (71.81)		
Q3 (20,001–40,000)	235 (23.93)	55 (23.40)	180 (76.60)		
Q4 (>40,000)	237 (24.13)	40 (16.88)	197 (83.12)		
**Smoking status**				10.867	0.004
Never smokers	670 (68.23)	145 (21.64)	525 (78.36)		
Former smokers	156 (15.89)	31 (19.87)	125 (80.13)		
Current smokers	156 (15.89)	52 (33.33)	104 (66.67)		
**Drinking status**				4.901	0.086
Never drinkers	624 (63.54)	142 (22.76)	482 (77.24)		
Former drinkers	126 (12.83)	22 (17.46)	104 (82.54)		
Current drinkers	232 (23.63)	64 (27.59)	168 (72.41)		
**Sports activities**				33.990	<0.001
No	442 (45.01)	141 (31.90)	301 (68.10)		
Yes	540 (54.99)	87 (16.32)	453 (83.68)		
**Self-reported health**				1.288	0.525
Healthy	290 (29.53)	74 (25.52)	216 (74.48)		
General	380 (38.70)	86 (22.63)	294 (77.37)		
Unhealthy	312 (31.77)	68 (21.79)	244 (78.21)		
**Distance to the village clinic (meters)**				3.615	0.164
≤100	238 (24.24)	65 (27.31)	173 (72.69)		
101–500	533 (54.28)	121 (22.70)	412 (77.30)		
>500		42 (19.91)	169 (80.09)		
**Multiple chronic diseases**				1.364	0.243
No	649 (66.09)	158 (24.35)	491 (75.65)		
Yes	333 (33.91)	70 (21.02)	263 (78.98)		
**Medications**				48.446	<0.001
No	169 (17.21)	74 (43.79)	95 (56.21)		
Yes	813 (82.79)	154 (18.94)	659 (81.06)		

For patients, awareness of blood pressure measurement was associated with household income per year, smoking status, sports activities and medications.

### Association Between Family Doctor Contracting Status and Awareness of Blood Pressure Measurement

Among 702 participants who had not signed a contract with family doctor (Table 2), 515 (73.36%) thought they would measure blood pressure regularly, and among 280 participants who had signed a contract, 239 (85.36%) had blood pressure measurement awareness. Table 2 indicates the statistical significances between family doctor contracting status and awareness of blood pressure measurement among hypertension patients (*P* < 0.001).

**Table 2:** The association between family doctor contracting status and blood pressure measurement awareness among hypertension patients in rural Shandong, China.

**Characteristics**	***N* (%)**	**Hypertension measurement awareness**		***x* ^2^**	***P*-value**
		**No [*N* (%)]**	**Yes [*N* (%)]**		
***N*** **(%)**	982 (100.00)	228 (23.22)	754 (76.78)		
**Family doctor contracting status**				16.156	<0.001
No	702 (71.49)	187 (26.64)	515 (73.36)		
Yes	280 (28.51)	41 (14.64)	239 (85.36)		

### Logistic Regression Analysis of Blood Pressure Measurement Awareness

The results of collinearity diagnosis showed that there was no multicollinearity among the independent variables (including gender, educational level, household income per year, smoking status, drinking status, self-reported health, distance to the village clinic, multiple chronic diseases, and medications), so the independent variables in the univariate analysis were included in the logistic regression model for adjusting.

In model 1 (Table 3), the association between contracting status and blood pressure measurement awareness was still significant (*P* < 0.001, *OR* = 2.075, 95% *CI* 1.391–3.095) after adjusting for the gender, education level, smoking status, drinking status, self-reported health, distance to the village clinic, multiple chronic diseases and medications. After the interaction items (age and contracting status) were included, model 2 showed that the interaction items were statistically significant (*P* = 0.042, *OR* = 1.747, 95% *CI* 1.020–2.992; *P* = 0.019, *OR* = 2.060, 95% *CI* 1.129–3.759). Meanwhile, sensitivity analysis was performed, as shown in the Supplementary Table.

**Table 3:** Logistic regression analysis of blood pressure measurement awareness among the hypertension patients in rural Shandong, China.

**Independent variables**	**Model 1 (no interaction)**			**Model 2 (age** **× contracting status)**		
	**OR**	**95% CI**	***P*-value**	**OR**	**95% CI**	***P*-value**
**Gender**
**Male (Ref.)**
Female	0.498	0.291–0.852	0.011	0.499	0.291–0.855	0.011
**Education level**
**Illiteracy (Ref.)**
Primary or below	0.901	0.603–1.345	0.609	0.887	0.592–1.327	0.559
Secondary school or above	1.010	0.627–1.626	0.968	0.995	0.617–1.605	0.985
**Household income per year (RMB, Yuan)**
**Q1 (≤8,400) (Ref.)**						
Q2 (8,401–20,000)	0.862	0.554–1.341	0.511	0.863	0.554–1.344	0.515
Q3 (20,001–40,000)	1.180	0.726–1.916	0.504	1.185	0.729–1.927	0.492
Q4 (>40,000)	1.737	1.038–2.906	0.035	1.764	1.052–2.956	0.031
**Smoking status**
**Never smokers (Ref.)**
Former smokers	0.656	0.366–1.177	0.157	0.659	0.367–1.181	0.161
Current smokers	0.439	0.261–0.740	0.002	0.439	0.260–0.739	0.002
**Drinking status**
**Never drinkers (Ref.)**
Former drinkers	1.111	0.566–2.179	0.760	1.119	0.571–2.193	0.744
Current drinkers	0.794	0.455–1.386	0.417	0.791	0.453–1.380	0.409
**Sports activities**
**No (Ref.)**
Yes	2.339	1.679–3.258	<0.001	2.338	1.679–3.257	<0.001
**Self-reported health**
**Healthy (Ref.)**
General	1.355	0.918–2.000	0.126	1.361	0.922–2.009	0.121
Unhealthy	1.591	1.033–2.450	0.035	1.608	1.043–2.477	0.031
**Distance to the village clinic (meters)**
**≤100 (Ref.)**						
101–500	1.308	0.897–1.906	0.163	1.302	0.893–1.898	0.170
>500	1.949	1.207–3.148	0.006	1.952	1.208–3.153	0.006
**Multiple chronic diseases**
**No (Ref.)**						
Yes	0.955	0.669–1.363	0.800	0.953	0.668–1.359	0.790
**Medications**
**No (Ref.)**						
Yes	3.291	2.250–4.815	<0.001	3.345	2.282–4.904	<0.001
**Age**
**<65 (Ref.)**						
≥65	0.884	0.612–1.277	0.511			
**Family doctor contracting status**
**No (Ref.)**						
Yes	2.075	1.391–3.095	<0.001			
**Age** **× Family doctor contracting status**
**Middle-aged** **× uncontracted (Ref.)**
Aged × uncontracted				0.816	0.543–1.226	0.327
Middle-aged × contracted				1.747	1.020–2.992	0.042
Aged × contracted				2.060	1.129–3.759	0.019

*95% CI, 95% confidence interval*.

**Figure 1:**
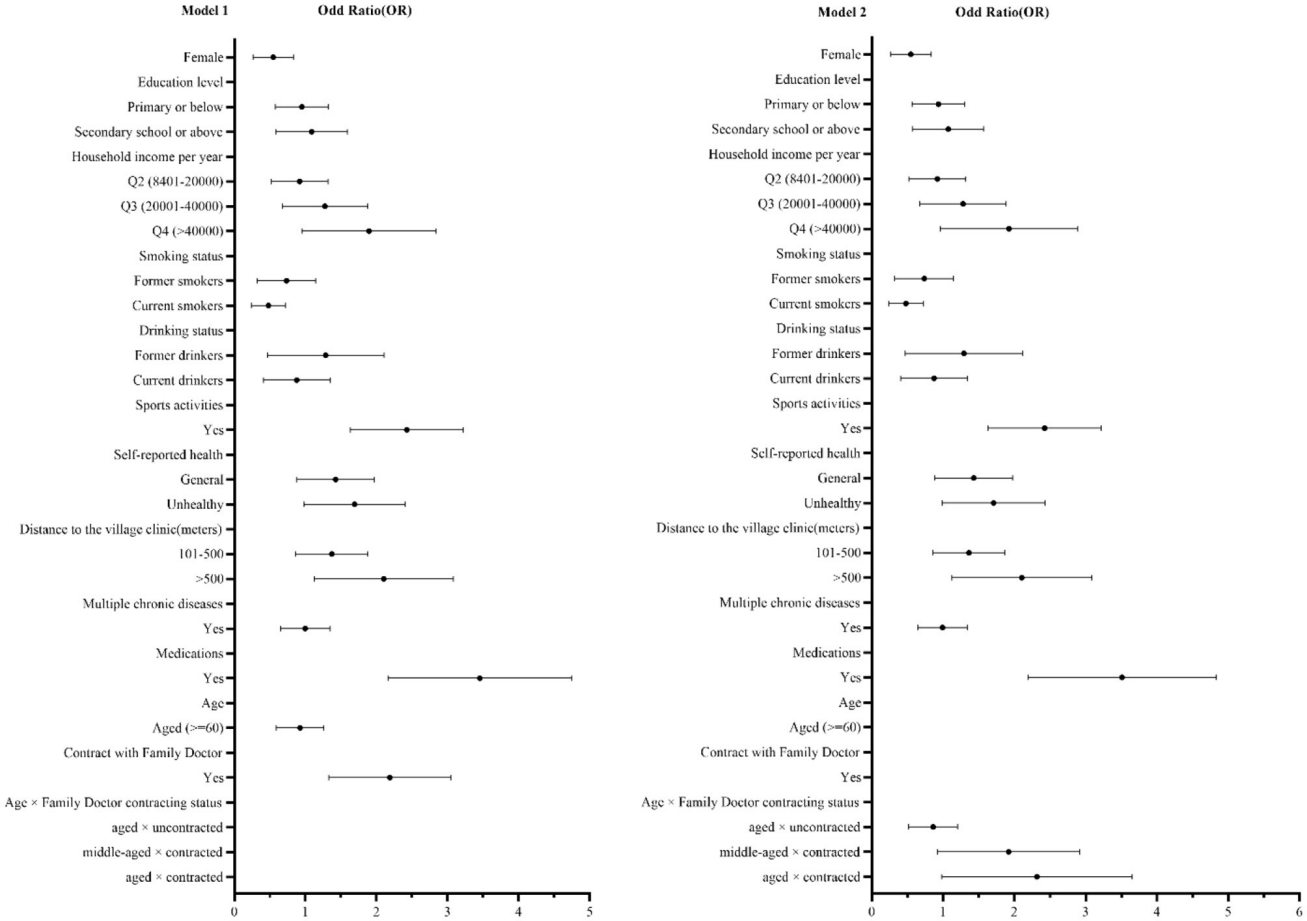
Forest plots of logistic regression analysis of blood pressure management awareness in rural Shandong, China.

In addition, the result showed that blood pressure measurement awareness was significantly associated with gender, household income, smoking status, sports activities, self-reported health, distance to the village clinic and medications for patients. Specifically, hypertension measurement awareness of women (*P* = 0.011, *OR* = 0.499, 95% *CI* 0.291–0.855) and smokers (*P* = 0.002, *OR* = 0.439, 95% *CI* 0.260–0.739) were significantly worse than men and never smokers. The higher the household income, the better the awareness of blood pressure measurement (*P* = 0.031, *OR* = 1.764, 95% *CI* 1.052–2.956). The awareness in patients with physical habits was significantly higher than that in patients without the habits (*P* < 0.001, *OR* = 2.338, 95% *CI* 1.679–3.257). Patients who considered themselves unhealthy had higher awareness of blood pressure measurement (*P* = 0.031, *OR* = 1.608, 95% *CI* 1.043–2.477). The farther the distance from the village clinic (*P* = 0.006, *OR* = 1.952, 95% *CI* 1.208–3.153), the better the awareness of blood pressure measurement. Awareness of blood pressure measurement was higher in patients taking hypertension medication (*P* < 0.001, *OR* = 3.345, 95% *CI* 2.282–4.904). As shown in Figure 1.

## Discussions

In the present investigation, we analyzed the association between family doctor contracting status and awareness of blood pressure measurement among Chinese hypertension patients. To our knowledge, this is the first study which reports the association between family doctor contracting status and blood pressure measurement awareness at different ages in China. The result showed a difference in the associations between family doctor contracting status and awareness of blood pressure measurement among hypertension patients at different ages.

In this study, the rate for awareness of blood pressure measurement among hypertension participants was ~76.8%. A study measuring patients' awareness of hypertension by asking “do you have blood pressure monitoring equipment at home?” found a rate of 33% (27), which was lower than this study. Health awareness were related to age, gender, educational level, social support, economic status and marital status (28), and the availability of home blood pressure monitoring equipment was affected by economic conditions. Some patients would choose to go to the village clinic for blood pressure measurement, so there was no home blood pressure monitoring equipment. Therefore, it was lower than the results of this study. The result suggested that hypertension residents should be encouraged to promote their awareness of blood pressure measurement.

The current study found that FDCSs were positively associated with the blood pressure measurement awareness of rural hypertension patients. The family doctors offered annual physical health examinations and four times of face-to-face health follow-up (6). These measures are important services for patients to increase their awareness of hypertension measurement. FDCSs-based hypertension management is critical for enhancing blood pressure measurement awareness of hypertension patients. To increase hypertension knowledge and awareness were closely associated with successful control of hypertension (19). In addition, most physicians thought that health education could influence blood pressure management awareness and lifestyle of hypertension patients to some extent (11). Long-term community management by family doctors for hypertension may increase the rate of management awareness, alter smoking and drinking habits, improve the rate and degree of blood pressure control, and reduce cardiovascular complications (29). The cross-sectional data of this study cannot infer the cause-effect relationship between family doctor contracting status and hypertension measurement awareness. However, we thought that good awareness of blood pressure measurement not only depended on its own health awareness, FDCSs was an important source of health information.

There was no statistical difference in blood pressure awareness between the middle-aged and the elderly who had not contracted with family doctors, but hypertension measurement awareness of patients who had contracted family doctors was better than those who had not signed. In contracted patients, awareness of aged adults was better than middle-aged adults. The results showed that FDCSs was an important source of health information for both middle-aged and old people. Compared with middle-aged adults, the health information of the older adults comes from a single source, uncontracted older adults lose an important source of health information. The elderly were more likely to be affected by hypertension. Physical discomfort and economic burden prompted the elderly patients to pay attention to the health education of the family doctors, and their health awareness was also enhanced (24). In addition, the elderly were also one of the priority population of family doctors' service. For middle-aged adults, on the one hand, the middle-aged were the main breadwinner of their families, so they knew the importance of their health, and family members would also pay attention to their health; on the other hand, middle-aged rural people were more likely to go out to work, so health education from family doctors was more important to them.

The present study also found that gender, smoking status, sport activities, household income, self-reported health, distance to village clinic and medications were important factors which were associated with awareness of blood pressure measurement. Women were often busy with housework, so they had fewer sources of information than men. According to their bad lifestyles, we speculated that the current smokers in this study may regard themselves as a healthier group or pay little attention to their health because of no symptoms, so they lacked awareness of blood pressure measurement. Patients with good health habits usually have good health awareness. Patients with higher family incomes were generally more concerned with improving quality of life and therefore had higher awareness of blood pressure measurement. The present study revealed that residents who reported their self-health as unhealthy had a higher blood pressure measurement awareness. Previous studies reported that residents who were aware they had high blood pressure reported poor health at a higher rate than those who were unconscious (30). Patients who has been taking hypertension medications for the last 2 weeks always pay close attention to change in their blood pressure, which depended on good awareness of blood pressure measurements.

In addition, we did not find the association between awareness of blood pressure measurement and education level, multiple chronic diseases. Regardless of the level of education, patients were concerned about their own health. Patients with low education level would actively acquire hypertension knowledge to make up for the knowledge difference brought by education level. Even if patients suffered from other diseases, they would pay attention to the measurement of hypertension, which was enough to show the importance of hypertension and the severity of its complications.

The findings in this study have some implications for the policy-making. The government should continuely to increase policy support to encourage residents to actively contract with family doctors and to improve as more as possible the related health measurement awareness, especially for uncontracted patients. Primary health care institutions should take efforts to strengthen health education and improve self-measurement awareness of residents, especially for uncontracted patients, female patients and patients with unhealthy life behaviors.

This study has several limitations. First, it was a cross-sectional study, therefore, the relationship between FDCSs and blood pressure measurement awareness cannot be interpreted as causal in nature. Second, regarding the chronic conditions, the detail information of blood pressure measurement awareness (such as the degree of awareness) and blood pressure control status were also unavailable, which were crucial to further understanding hypertension patient's management. Finally, this study was only conducted in Shandong province and the generalizability of the main findings needs to be verified in China.

## Conclusions

The study demonstrated a significant association between FDCSs and blood pressure management awareness among hypertensive patients in China. Compared with non-contracted patients, patients with FDCSs reported higher hypertension management awareness. And contracted patients with different age groups had different awareness of blood pressure measurement. Some other factors were also found to be associated with blood pressure management awareness among hypertension patients in rural Shandong, including gender, smoking status, sports activities, household income, self-reported health, distance to the village clinic and medications. In order to improve the blood pressure management awareness of hypertensive patients, village clinics and township hospitals should take efforts to strengthen publicity and education based on FDCSs, especially for non-contracted, middle-aged, female patients and patients with unhealthy life behaviors.

## Data Availability Statement

The raw data supporting the conclusions of this article will be made available by the authors, without undue reservation.

## Author Contributions

CY and CZ designed the study, CY selected and processed the data and wrote the manuscript. YY, DZ, and JL (4th author) revised the manuscript. All authors contributed to the subsequent drafts, reviewed, and endorsed the final submission.

## Funding

This study was supported by the National Science Foundation of China (71974117, 71774104, and 71473152), the China Medical Board (16–257), Cheeloo Youth Scholar Grant, Shandong University (IFYT1810, IFYT181031), and NHC Key Laboratory of Health Economics and Policy Research (Shandong University, NHC-HEPR2019001). The funding bodies had no role in the design, data collection, analysis, interpretation of the data, and writing of this article.

## Conflict of Interest

The authors declare that the research was conducted in the absence of any commercial or financial relationships that could be construed as a potential conflict of interest.

## Publisher's Note

All claims expressed in this article are solely those of the authors and do not necessarily represent those of their affiliated organizations, or those of the publisher, the editors and the reviewers. Any product that may be evaluated in this article, or claim that may be made by its manufacturer, is not guaranteed or endorsed by the publisher.
