# Supplementary material for: Family Doctor Contract Services and Awareness of Blood Pressure Measurement Among Hypertension Patients: A Cross-Sectional Study in Rural Shandong, China
Source: Front Public Health. 2022 Mar 16;10:757481. doi: 10.3389/fpubh.2022.757481 (PMC8966041; doi:10.3389/fpubh.2022.757481)
Supplement: Supplementary file 1 [file Data_Sheet_1.docx]

Supplementary Material

# Supplementary Figures and Tables

## Sensitivity analysis

To further verify the correlation between family doctor contracting services and blood pressure awareness at different ages, stratified analysis and logistic regression were used.

**Table 1. Sensitivity analysis of blood pressure management awareness among the hypertension patients in Shandong, China.**

| **Independent variables** | **Model 1(total)** | | | **Model 2(middle-aged)** | | | **Model 3(aged)** | | |
| --- | --- | --- | --- | --- | --- | --- | --- | --- | --- |
|  | **OR** | **95%CI** | ***p*-value** | **OR** | **95%CI** | ***p*-value** | **OR** | **95%CI** | ***p*-value** |
| **Gender** |  |  |  |  |  |  |  |  |  |
| Male(Ref.) |  |  |  |  |  |  |  |  |  |
| Female | 0.507 | 0.298-0.865 | 0.013 | 0.457 | 0.214-0.979 | 0.044 | 0.493 | 0.219-1.112 | 0.088 |
| **Education level** |  |  |  |  |  |  |  |  |  |
| Illiteracy(Ref.) |  |  |  |  |  |  |  |  |  |
| Primary or below | 0.918 | 0.618-1.365 | 0.674 | 1.198 | 0.655-2.194 | 0.558 | 0.714 | 0.402-1.268 | 0.251 |
| Secondary school or above | 1.058 | 0.671-1.668 | 0.808 | 1.243 | 0.661-2.337 | 0.499 | 0.910 | 0.404-2.051 | 0.821 |
| **Household income per year(RMB, Yuan)** | | | | |  |  |  |  |  |
| Q1(<8400)(Ref.) |  |  |  |  |  |  |  |  |  |
| Q2(8401-20000) | 0.888 | 0.576-1.369 | 0.591 | 0.899 | 0.415-1.946 | 0.787 | 0.780 | 0.439-1.383 | 0.395 |
| Q3(20001-40000) | 1.236 | 0.777-1.968 | 0.371 | 1.015 | 0.472-2.181 | 0.970 | 1.673 | 0.774-3.620 | 0.191 |
| Q4(>40000) | 1.813 | 1.101-2.986 | 0.019 | 1.848 | 0.827-4.128 | 0.134 | 1.510 | 0.704-3.239 | 0.290 |
| **Smoking status** |  |  |  |  |  |  |  |  |  |
| Never smokers(Ref.) | |  |  |  |  |  |  |  |  |
| Former smokers | 0.656 | 0.365-1.177 | 0.158 | 1.358 | 0.564-3.273 | 0.495 | 0.356 | 0.153-0.829 | 0.017 |
| Current smokers | 0.442 | 0.262-0.745 | 0.002 | 0.686 | 0.330-1.429 | 0.315 | 0.298 | 0.133-0.666 | 0.003 |
| **Drinking status** |  |  |  |  |  |  |  |  |  |
| Never drinkers(Ref.) | |  |  |  |  |  |  |  |  |
| Former drinkers | 1.095 | 0.559-2.145 | 0.791 | 0.602 | 0.216-1.680 | 0.332 | 2.046 | 0.805-5.200 | 0.133 |
| Current drinkers | 0.792 | 0.454-1.382 | 0.411 | 0.351 | 0.157-0.786 | 0.011 | 1.660 | 0.712-3.868 | 0.240 |
| **Sports activities** |  |  |  |  |  |  |  |  |  |
| No(Ref.) |  |  |  |  |  |  |  |  |  |
| Yes | 2.315 | 1.665-3.219 | <0.001 | 2.099 | 1.326-3.324 | 0.002 | 2.854 | 1.732-4.704 | <0.001 |
| **Self-reported health** |  |  |  |  |  |  |  |  |  |
| Healthy(Ref.) |  |  |  |  |  |  |  |  |  |
| General | 1.349 | 0.915-1.990 | 0.131 | 1.382 | 0.815-2.346 | 0.230 | 1.503 | 0.815-2.774 | 0.192 |
| Unhealthy | 1.585 | 1.030-2.439 | 0.036 | 1.552 | 0.851-2.830 | 0.151 | 1.730 | 0.898-3.335 | 0.102 |
| **Distance to the village clinic(meters)** | | |  |  |  |  |  |  |  |
| <100(Ref.) |  |  |  |  |  |  |  |  |  |
| 101-500 | 1.312 | 0.901-1.912 | 0.157 | 1.622 | 0.954-2.757 | 0.074 | 1.027 | 0.584-1.806 | 0.926 |
| >500 | 1.947 | 1.206-3.143 | 0.006 | 1.598 | 0.838-3.047 | 0.154 | 2.890 | 1.342-4.566 | 0.007 |
| **Multiple chronic diseases** | |  |  |  |  |  |  |  |  |
| No(Ref.) |  |  |  |  |  |  |  |  |  |
| Yes | 0.947 | 0.664-1.350 | 0.764 | 0.620 | 0.372-1.035 | 0.067 | 1.412 | 0.839-2.374 | 0.194 |
| **Medications** |  |  |  |  |  |  |  |  |  |
| No(Ref.) |  |  |  |  |  |  |  |  |  |
| Yes | 3.257 | 2.230-4.757 | <0.001 | 3.947 | 2.399-6.495 | <0.001 | 2.629 | 1.383-4.997 | 0.003 |
| **Family doctor contracting status** | | |  |  |  |  |  |  |  |
| No(Ref.) |  |  |  |  |  |  |  |  |  |
| Yes | 2.063 | 1.385-3.075 | <0.001 | 1.806 | 1.042-3.129 | 0.035 | 2.476 | 1.342-4.566 | 0.004 |

## Questionnaire

**Q1**. What's your gender?

1=male, 2=female

**Q2**. The year of your birth (e.g., 1975) ______

**Q3**. What is your highest level of education?

1= illiteracy, 2= nursery/kindergarten, 3= primary school, 4= junior high school, 5= senior high school, 6= technical secondary school, 7= junior college, 8= university, 9= master, 10= doctor

**Q4**. How much is your total income (gross income) in 2017 (i.e. farming, forestry, animal husbandry, fishing; Working and wage income; Income from wholesale and retail trade, catering, transportation; Subsidies and gifts from friends and relatives; Value of unsold products, property income, transfer income)? _____yuan

**Q5**. How do you think of your physical health now?

1= very good, 2= very good, 3= general, 4= poor, 5= very poor

**Q6.** Does anyone in your family suffer from high blood pressure?

1=yes, family number code_____ 2=no

**Q7**. Does anyone in your family suffer from diabetes?

1=yes, family number code_____ 2=no

**Q8**. Does anyone in your family suffer from coronary artery disease?

1=yes, family number code_____ 2=no

**Q9.** Have you taken any hypertension medication in the last two weeks?

1=yes, 2=no

**Q10.** Do you think you need to measure your blood pressure regularly?

1=yes, 2=no

**Q11.** Do you smoke?

1= Never smoking, 2= I used to smoke, but now I quit, 3= current smoker

**Q12.** Have you been drinking alcohol?

1= Never drink, 2= I used to drink, but I quit, 3= current drinker

**Q13***.* Have you participated in any sports activities (except farm work) in the past month?

1=yes, 2=no

**Q14**. How far is your home from the village clinic? _____meters

**Q15**. Did you contract with the family doctors this year?

1=yes, 2= no
